# Molecular Dynamics Simulations of Amylose- and Cellulose-Based Selectors and Related Enantioseparations in Liquid Phase Chromatography

**DOI:** 10.3390/molecules28217419

**Published:** 2023-11-03

**Authors:** Roberto Dallocchio, Alessandro Dessì, Barbara Sechi, Paola Peluso

**Affiliations:** Unit of Enantioselective Chromatography and Molecular Recognition, Institute of Biomolecular Chemistry ICB, Secondary Branch of Sassari, CNR, Traversa La Crucca 3, Regione Baldinca, Li Punti, 07100 Sassari, Italy; roberto.dallocchio@cnr.it (R.D.); alessandro.dessi@cnr.it (A.D.); barbara.sechi@cnr.it (B.S.)

**Keywords:** computational methods, enantioselective recognition, enantioseparation, molecular dynamics, polysaccharide-based selectors

## Abstract

In the last few decades, theoretical and technical advancements in computer facilities and computational techniques have made molecular modeling a useful tool in liquid-phase enantioseparation science for exploring enantioselective recognition mechanisms underlying enantioseparations and for identifying selector–analyte noncovalent interactions that contribute to binding and recognition. Because of the dynamic nature of the chromatographic process, molecular dynamics (MD) simulations are particularly versatile in the visualization of the three-dimensional structure of analytes and selectors and in the unravelling of mechanisms at molecular levels. In this context, MD was also used to explore enantioseparation processes promoted by amylose and cellulose-based selectors, the most popular chiral selectors for liquid-phase enantioselective chromatography. This review presents a systematic analysis of the literature published in this field, with the aim of providing the reader with a comprehensive picture about the state of the art and what is still missing for modeling cellulose benzoates and the phenylcarbamates of amylose and cellulose and related enantioseparations with MD. Furthermore, advancements and outlooks, as well as drawbacks and pitfalls still affecting the applicability of MD in this field, are also discussed. The importance of integrating theoretical and experimental approaches is highlighted as an essential strategy for profiling mechanisms and noncovalent interaction patterns.

## 1. Introduction

In the last decade, the use of molecular modeling has become rather popular in liquid-phase enantioseparation science. In this context, several studies integrated experimental and computational/theoretical analysis to disclose the molecular bases of the mechanisms underlying the enantioseparation process. For this purpose, quantum mechanics (QM) calculations, molecular docking, and molecular dynamics (MD) are the main techniques used for exploring enantioseparation at molecular levels, which involves attempting to deconvolute mechanisms and related noncovalent interactions [1,2,3,4,5]. The objective is ambitious, but it would significantly feed back into enantioseparation science. Indeed, a full understanding of the enantioseparation at molecular level would allow analytical scientists to tackle the enantioseparation task on a rational basis, abandoning classical trial-and-error approaches. This is still a hot topic of urgency for the rational application of polysaccharide derivatives and for designing new polymers as chiral selectors in liquid-phase chromatography.

**Figure 1:**
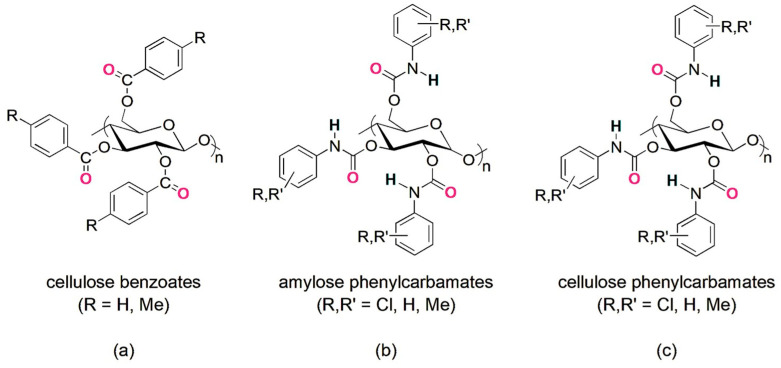
Drawing structures of cellulose benzoates (**a**) and phenylcarbamates of amylose (**b**) and cellulose (**c**).

The use of oligo- and polysaccharides as chiral selectors for liquid-phase enantioseparations dates back to the first decades of the 1900s [6]. In the beginning, the enantiorecognition performances of native and underivatized oligo- and polysaccharides were not very successful, because, likely, the presence of multiple hydroxyl groups as strong hydrogen bond (HB) donors and leading recognition sites was not effective for the enantioseparation of different classes of chiral compounds. Indeed, the interaction system of underivatized oligo- and polysaccharides was based on strong HBs as leading interactions and, thus, less prone to work successfully for the enantiorecognition of chiral compounds not containing HB sites. Furthermore, weak noncovalent interactions and HBs of medium strength are, in general, more effective than strong HBs for efficiently modulating subtle energetic differences between the transient diastereomeric complexes, thus improving and optimizing enantioseparation. To surpass the limitations of native polysaccharides, Okamoto’s group designed and prepared cellulose benzoates and phenylcarbamates of cellulose and amylose in the 1980s, featuring one or two electron-donor or electron-withdrawing substituents on the aryl ring [7,8,9,10,11]. Later, in the 1990s, polymers containing substituents of a different nature (methyl and chlorine) were also developed to generate finely tuned selectors in terms of HB strength [12,13,14]. In the last forty years, these polymeric selectors have shown unsurpassed performances for the enantioseparation of chiral compounds [15,16,17]. Today, several chiral columns based on cellulose benzoates and phenylcarbamates of amylose and cellulose (Figure 1) are commercially available, and these contain the polymeric selector physically adsorbed on or covalently immobilized to a silica support [18].

**Figure 2:**
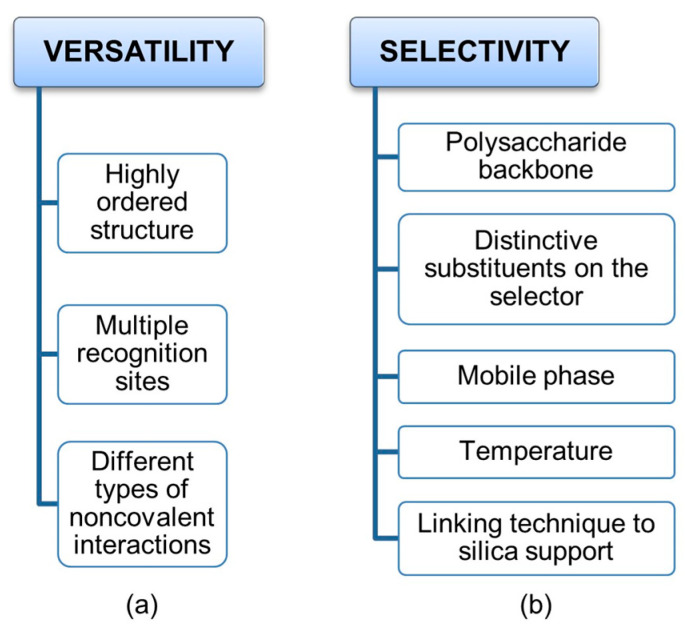
Factors affecting the versatility (**a**) of polysaccharide-based selectors and their selectivity (**b**) toward chiral compounds.

On the one hand, a highly ordered secondary structure and the presence of multiple recognition sites, which are able to exert different types of noncovalent interactions, determine the versatility of cellulose benzoates and phenylcarbamates of amylose and cellulose as privileged platforms for enantioseparation (Figure 2a). On the other hand, this structural complexity makes the mechanisms acting at molecular levels rather challenging to deconvolute, because multiple noncovalent interactions can contribute co-operatively or anti-cooperatively to the analyte–selector interaction. Furthermore, boundary conditions are particularly important to address, control, and optimize enantioseparations promoted by polysaccharide-based chiral stationary phases (CSPs) (Figure 2b). Indeed, these CSPs can function equally well with hydrocarbon-based mixtures, polar organic solvents, aqueous-based mixtures, and under hydrophilic interaction liquid chromatography conditions. In fact, changing polarity and molecular structure of mobile phase components allows for the fine tuning of the noncovalent interactions involved in binding and recognition, given that solvent molecules may competitively interact with analyte and selector surfaces and with analyte–selector complexes favoring some analyte–selector contacts but disfavoring others. Temperature may also impact enantioseparation and, for certain analytes showing entropy-driven thermodynamics, enantioselectivity increases with increasing temperature. Moreover, a phase transition of polysaccharide-based chiral selectors was observed even at 30–40 °C [19,20,21].

On these bases, it is evident that several factors like structure of analyte and selector, mobile phase polarity, and temperature along with the technique used to fix the polymer on the silica surface, coating or covalent immobilization, may contribute to the enantioseparation extent. Thus, in principle, the virtual model of an enantioseparation should account for all these factors. In practice, this is not a trivial issue. QM calculations are, in general, unsuitable for the study of large complex molecular systems like liquid-phase chromatographic systems because of the prohibitive computational cost required for studying comprehensively large solvated molecular environments. Thus, although QM-based studies successfully contributed to the understanding of some aspects of the mechanisms acting within polysaccharide-based selectors [22,23], these studies may be exclusively carried out on analytes and on small portions of the selector. Molecular docking is very popular in liquid-phase enantioseparation [24,25,26], but this technique presents at least two pitfalls, given that in most cases, (*a*) the dynamic nature of the chromatographic enantiorecognition process is not considered, and (*b*) the simulation is performed in the vacuum, neglecting solvation effects. Otherwise, MD paves the way to comprehensively model the liquid-phase enantioseparation system, accounting for the dynamics of the enantioseparation process and explicitly accounting for solvation effects.

Given the importance of the field, this review aims to provide the reader with a comprehensive picture about the state of the art and what is still missing for MD simulations of cellulose benzoates and phenylcarbamates of amylose and cellulose as well as related enantioseparation processes. For this purpose, after a short description of the structural features of polysaccharide benzoates and phenylcarbamates and of the bases of MD as a simulation technique, this paper presents a systematic analysis of the MD simulations performed in the field until now. Furthermore, the advancements and outlooks, as well as drawbacks and pitfalls that still affect the applicability of MD to model enantioseparations promoted by polysaccharide-derivatives, are also discussed.

## 2. Polysaccharide-Based Selectors

**Figure 3:**
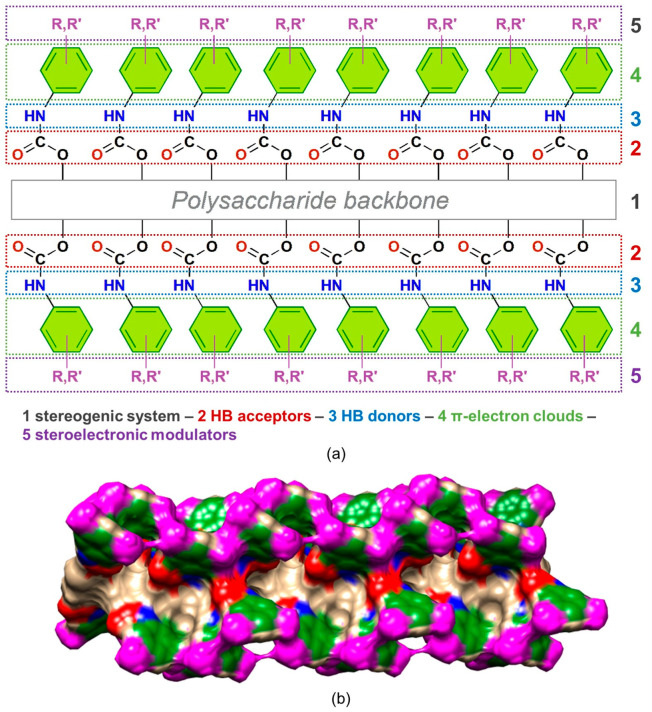
Structure of polysaccharide phenylcarbamates: (**a**) bidimensional drawing structure highlighting the modular organization of the polymer derivative (R,R′=Cl, H, methyl); (**b**) electron density surface of an amylose *tris*(3,5-dimethylphenylcarbamate) (ADMPC) dodecamer (12-mer), graphically generated by using Chimera 1.13.1 [31]. Colors: Aryl group (green), N-H (blue), C=O (red), methyl group (magenta).

Polysaccharide phenylcarbamates are characterized by a modular structure consisting of five pivotal regions, each with specific functions that, when combined, contribute synergistically to promote the recognition of the enantiomers of a chiral compound (Figure 3a).

The polysaccharide backbone (1) comprises the stereogenic regions formed by D-glucopyranosyl residues bonded by α- (amylose) or β- (cellulose) (1,4)-glycosidic linkages. Due to these specific glycosidic linkages featuring polysaccharide chains, amylose and cellulose derivatives are characterized by a conformationally chiral (helical) secondary structure. In the 1980s–1990s period, a left-handed threefold 3/2 helix was observed for the cellulose *tris*(phenylcarbamate) (CTPC) via X-ray diffraction (XRD) analysis and molecular modeling [27,28]. In 2002, Okamoto et al. proposed a left-handed 4/3 helical structure as the most probable for amylose *tris*(3,5-dimethylphenylcarbamate) (ADMPC) based on 2D NOESY spectroscopy studies and computational modeling [29].

Each glucopyranosyl residue presents three phenylcarbamate pendant groups introduced in the native polysaccharide by functionalizing the hydroxyl groups at the 2, 3, and 6 glucopyranosyl positions. On this basis, in the inner part of the polymer groove, two adjacent regions contain carbonyl oxygen atoms (2) and amidic hydrogen atoms (3) as HB acceptors and HB donors, respectively. Thus, the carbamate sites can exert polar intermolecular noncovalent interactions, like HBs, dipole–dipole interactions, halogen and chalcogen bonds with analyte enantiomers, and intramolecular HBs, significantly contributing to the highly ordered secondary structure of the polymer and assuring the uniformity of the adsorption sites [6,17]. Furthermore, at the periphery of the polymer groove, a hydrophobic region consisting of aromatic rings (4) exists, which may exert π–π and π–H interactions. The substituents, methyl or/and chlorine, of the phenylcarbamate pendant groups (5) act as stereoelectronic modulators toward the carbamate moieties, impacting the performances of the corresponding polysaccharide derivatives as chiral selectors. Experimental and theoretical studies [2,14,30] demonstrated that these substituents exert an opposite effect on the two main carbamate recognition sites, C=O and N-H. Thus, whereas the methyl group increases the nucleophilic (HB acceptor) ability of the carbonyl oxygen atoms and decreases the electrophilic properties of the amidic hydrogen atoms as HB donors, the chlorine increases the electrophilic ability of the amidic hydrogen atoms and decreases the nucleophilic properties of the carbonyl oxygen atoms. Overall, the presence of regions 2–4 generates the expansion of the native polymer into a direction that is perpendicular to the axis of the polysaccharide backbone, providing an extended surface (Figure 3b) [31], which can interact with a chiral molecule, differentiating its enantiomers [2,32].

Cellulose benzoates feature similar structures, but the region containing the N-H groups as HB donors is missing in these chiral selectors.

It is worth mentioning that other polysaccharide derivatives have also been evaluated as CSPs. Recently, chitin and chitosan were used as polysaccharide backbones for the development of chiral selectors [33,34]. However, no chiral column based on these polysaccharides was commercialized so far.

## 3. Molecular Dynamics: A Short Description of the Technique

**Figure 4:**
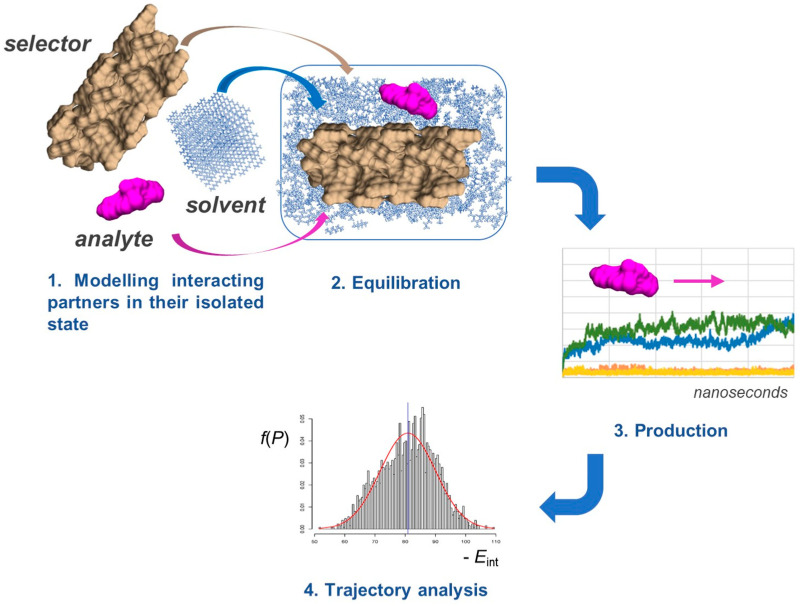
General workflow for MD simulations.

MD offers a unique opportunity to model how the enantiomers of a chiral compound move and interact over time on a selector surface (Figure 4). This approach is based on classical mechanical equations of motion applied to the enantioseparation system to simulate contact between an enantiomer and selector as interacting particles [35], accounting for their interaction with solvent molecules as well.

Commonly used programs for MD simulations include AMBER [36], CHARMM [37], Desmond [38], GROMACS [39], and LAMMPS [40], among others. Currently, available tools allow for the quantifying of the energetic and geometrical features of the process and their statistical distribution over MD production time.

In a typical MD experiment, the molecular system runs over the production time. In 1995, Lipkowitz stated that “*Simulation time periods are typically in the picosecond (10^−12^ s) range*” [41]; today, MD can be carried out over a time ranging from ten to hundreds of nanoseconds (ns), even microseconds [42]. However, it is worth specifying that, for application in enantioselective recognition, MD predominantly remains in the nanosecond domain. The process is iterated in thousands of steps to bring the system to an equilibrium state, acquiring information about the atomic positions, velocities, and other variables as a function of time. This set of data emerging from the MD experiment is the “trajectory”, and equilibrium and dynamic properties of the enantioseparation system can be calculated from the trajectory data set. The root mean square deviation (RMSD) of all atoms in a molecule can be plotted against time to summarize the degree of fluctuation for the entire structure. For instance, by comparing the RMSD values, a lower degree of fluctuation of the analyte–selector system was found for amylose derivatives compared to the cellulose-based polymers, confirming the more compact structure of the amylose-based groove [43].

In this review, we will not discuss the chemo-physical and mathematical details of MD in depth; just some essential equations of practical usefulness will be mentioned. Based on the trajectory data set, given the selector and the enantiomers of a chiral analyte, the interaction energy (*E*_int_) between enantiomer and selector can be calculated based on the energies of the selector–enantiomer complex, the selector, and the enantiomer (Equation (1)):

*E*_int_ = *E*_total_ − *E*_analyte_ − *E*_selector_ (1)

where the *E*_int_ term is derived from the contributions of the van der Waals (vdW) and the electrostatic (el) interaction terms (Equation (2)):

*E*_int_ = *E*_el_ + *E*_vdW_ (2)

The most popular force fields include dispersion interactions through the Lennard-Jones potential equation (Equation (3)):

(3) $$E_{LJ,ij}(r_{ij})=\frac{A_{ij}}{r_{ij}^{12}}-\frac{C_{6,ij}}{r_{ij}^{6}}$$

where the *A*/*r*^12^ term of this equation represents Pauli repulsion, whereas dispersion interactions are represented by the *C*_6_/*r*^6^ term.

As mentioned above, the mobile phase has a pivotal role in enantioseparation; thus, solvent parametrization can strongly influence the energy of the different (diastereomeric) complex conformations observed in MD. The solvent can be parametrized by treating it explicitly or implicitly [44]. When the explicit treatment is used, solvent molecules are introduced in the virtual system by computing interactions involving solvent atoms. On the contrary, implicit solvent methods approximate the solvent medium to a continuum. In fact, although this method accelerates simulations by significantly reducing the number of particles in the system, the evaluation of solvent effects becomes less reliable. The possibility of treating a solvent explicitly is one of the major advantages of MD compared to molecular docking. 

The main issue in the application of MD to processes promoted by polysaccharide-based selectors concerns the lack of three-dimensional structures defined experimentally by XRD for most amylose- and cellulose-based selectors, and this absence of benchmark structures makes the virtual simulation challenging. 

## 4. Application of MD to Model Polysaccharide-Based Selectors

The first attempts to model polysaccharide-based selectors date to the period between the end of the 1980s and early 1990s [41,45]. Over time, a limited number of polysaccharide-based selectors have been modeled with MD (Table 1). Until now, the possibility of modeling these structures depended on the availability of XRD structures or reliable structural information derived from spectroscopic analysis.

**Figure 5:**
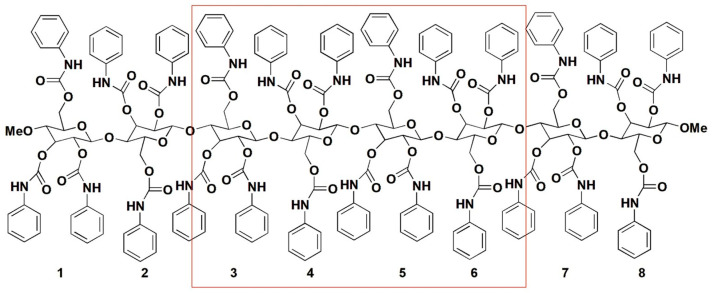
Octamer of CTPC.

**Figure 6:**
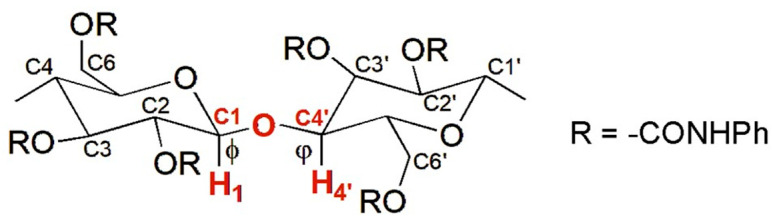
The glycosidic linkage in cellulose-based derivatives.

In 1995, Okamoto et al. reported the modeling of CTPC (**mod. 1**) [28]. This derivative was selected because its structure was known, determined by Vogt and Zugenmaier in 1985 through XRD of a CTPC fiber [27]. The optimization of the CTPC structure was not entirely performed by MD in this case, but the simulation technique was used to refine the virtual model. Indeed, the overall model preparation consisted of the following steps: (*a*) full energy molecular mechanics (MM) minimization of a monomer of CTPC, containing methoxyl groups at the 1- and 4-positions of the glucopyranosyl unit; (*b*), construction of an octamer (8-mer) (Figure 5) with a left-handed threefold (3/2) helix according to the XRD structure of CTPC by using optimized units. The dihedral angles defined by H_1_-C_1_-O-C_4′_ (*ϕ*) and H_4′_-C_4′_-O-C_1_ (*φ*) were 60° and 0°, respectively (Figure 6). It is worth mentioning that these values remain benchmark geometrical parameters for the construction of cellulose-based models; (*c*) optimization of the 8-mer CTPC by the steepest descents method, obtaining a metastable structure featuring HBs between the NH protons of the carbamate moieties at the 6-positions and the carbonyl oxygens at the 2-positions, with 2.634 Å as HB length; (*d*) application of an MD simulation to the optimized 8-mer of CTPC by using the CHARMM force field [37], and extraction of the structures with lower energies from the trajectory files; and (*e*) MM calculation of the extracted structures. Given that, the authors did not observe significant changes in the CTPC model after the MD simulation, and the modeled structure was almost similar to the XRD structure. In particular, the same motif with the pendant groups at the 2- and 3-positions and that at the 6-position of the neighboring glucopyranose unit close to each other could be observed in both structures.

On the other hand, some differences also occurred between calculated and experimental models: (*a*) the modeled structure was not exactly a 3/2 helix; (*b*) the rotation angles around each C_1_–C_4_ axis were in the range 110–113°, depending on the position of each glucopyranose unit within the 8-mer; and (*c*) conformation between the carbonyl group and the ether oxygen of the pendant group at the 2-position was *s*-*cis*, whereas those at the 3- and 6-positions were *s*-*trans*. The latter was the main difference between modeled and XRD structures, which presented all the conformations as *s*-*trans*. It is worth mentioning that, as reported by the authors, the conformation of the starting 8-mer before minimization was *s*-*trans*. Thus, the conformation at the 2-position changed to *s*-*cis* during minimization.

Later, Okamoto et al. applied a similar method by using MM calculations and picosecond MD simulations for modelling CTPC (**mod. 3**) and cellulose *tris*(3,5-dimethylphenylcarbamate) (CDMPC) (**mod. 4**) [47] as nonamers (9-mer). The optimized structures of CTPC and CDMPC showed a similar left-handed 3/2 helix with glucose residues regularly arranged along the helical axis. However, likely due to the steric hindrance exerted by the methyl groups on the phenyl groups, the aromatic rings of CDMPC appeared to be arranged differently compared to the CTPC.

These models were used to explore the interaction modes between CTPC and CDMPC and chiral compounds by using MM calculations to obtain low energy selector/analyte complexes [28,47]. For this purpose, *trans*-stilbene oxide (**1**), *trans* 1,2-diphenylcyclopropane (**2**), and benzoin (**3**) were used as test probes, considering the chromatographic enantioseparations of these analytes with polysaccharide phenylcarbamate-based selectors as benchmark experimental data.

**Figure 7:**
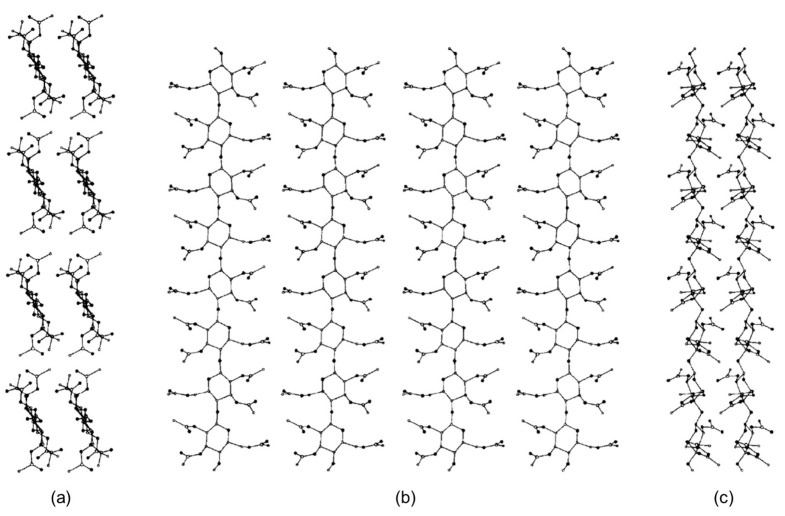
The nanocrystallite of cellulose triacetate (CTA). Views are projected onto the (**a**) *a*–*b* plane, (**b**) *a*–*c* plane, and the (**c**) *b*–*c* plane (Reproduced with permission from ref. [46]. Copyright 1997, Elsevier).

In 1997, Peterson and Lipkowitz carried out stochastic 300 ps MD on a nanocrystallite of cellulose triacetate (CTA) (**mod. 2**) [46]. The main question the authors posed in their research was “*what are the structural features of the surface layer of a cellulose triacetate layer used in chiral chromatography?*”. To respond to this question, a multistrand 8-mer CTA model (Figure 7) was constructed from a cellobiose unit in a parallel-strand unit cell as calculated by Wolf et al. [57]. On this basis, four cellobiose units were made into a strand with a (fiber) repeat distance of 10.5 Å. Four of these strands were placed side by side in parallel with a separation of 12 Å to construct the *a*–*c* face of the CTA virtual crystal. A second, duplicate layer was placed 5.2 Å below the first. The authors remarked that, to make the study computationally feasible, the following restrictions and approximations were applied: (*a*) the solvent was not represented; (*b*) only a single subsurface layer was included in the simulation to represent the bulk lattice underpinning the surface layer; (*c*) in the subsurface layer methine, methylene and methyl groups were treated as united atoms, while polar hydrogens were explicitly included; (*d*) terminal oxygen atoms were kept as hydroxyl groups instead of acetyl groups; (*e*) the simulations were performed on a small portion of an actual CTA microcrystallite.

Based on this study, the overall structural features of the CTA I surface appeared to be lattice-like. The authors observed that rolling motions of glucopyranose rings allowed for cavity formation able to host small molecules, exclusively. The acetyl side chains were shown to be very flexible, which signified that they could bind to many analyte molecules in a wide range of orientations. While the study was focused on the structural and dynamical features of the CTA I surface to profile its relative flexibility, it did not explore how enantiodifferentiation took place.

In 2007, Wirth et al. constructed the ADMPC polymer [48], starting from the 12-mer of ADMPC (**mod. 5**) released by Okamoto et al. and developing based on spectroscopic structural information [29]. For this structure, the dihedral angles defined by H_1_-C_1_-O-C_4′_ (ϕ) and H_4′_-C_4′_-O-C_1_ (φ) were −68.5° and −42.0°, respectively. For the ADMPC, **mod. 5** remains a seminal benchmark model in this field, referred to by most studies performed in the last two decades.

To eliminate finite size effects, which could result by using a relatively short polymer chain, Wirth et al. created a chain of infinite length using periodic boundary conditions [48]. Through this “trick”, the finite size effect was removed without including many atoms. In this study, the polysaccharide backbone atoms were fixed to the positions determined by Okamoto’s group through nuclear magnetic resonance (NMR) analysis [29], whereas the phenyl side chains were allowed to move freely. Then, the structure was minimized with the COMPASS force field, and “quenched annealing” was used to reduce the possibility of a local minimum, following the following workflow: (*a*) an initial equilibration MD run for 100 ps at 500 K was performed to allow the molecules to overcome energy barriers to fully explore the potential energy hypersurface; (*b*) a production run of 200 ps was then carried out, where a frame was saved every 10000 steps, thus generating 20 frames, each of which was subsequently minimized to a convergence criterion of 0.1 kcal·mol^−1^·Å^−1^, removing any unphysical geometries and finding the nearest minimum-energy structure.

**Figure 8:**
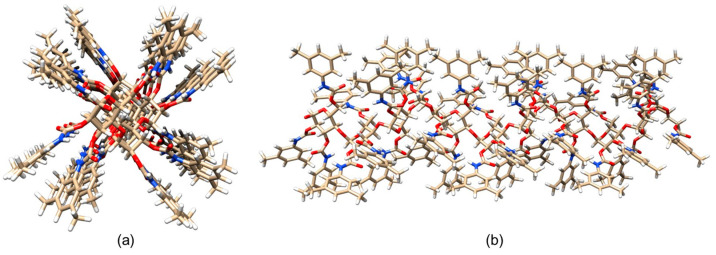
A three-dimensional structure of ADMPC oligomer (12-mer): (**a**) viewpoint along the chain axis, (**b**) viewpoint perpendicular to the chain axis. Colors: carbon (tan), hydrogen (grey), nitrogen (blue), oxygen (red) (Reproduced with permission from ref. [48]. Copyright 2007, American Chemical Society).

The lowest energy structure (Figure 8) was used for MD simulations of enantioseparation processes (see Section 5).

In 2008, Franses et al. studied ADMPC (**mod. 6**), amylose *tris*((*S*)-α-methylbenzylcarbamate) (ASMBC) (**mod. 7**), and CDMPC (**mod. 8**) by using different experimental techniques and MD [22,58]. For ADMPC and ASMBC, 12-mer models with four-fold helixes were constructed, whereas for CDMPC, a 9-mer model with a three-fold helix was prepared. The ADMPC and CDMPC structures were consistent with the models previously proposed by Okamoto’s group [28,29,48]. The rod diameters and helical pitches of the polymers, as geometrical parameters, were determined using XRD and then incorporated into the MD calculations. The XRD results showed that the helical pitches for the two amylose-based polymers were the same, 14.6 Å, but the interrod packing distance was different, 18.9 Å for ADMPC versus 16.9 Å for ASMBC. For CDMPC, the pitch, 16.2 Å, and the packing distance, 21.0 Å, were significantly larger than those of the other two polymers. On the other hand, based on attenuated total reflection infrared spectroscopy (ATR-IR) analyses, the CDMPC cavities appeared slightly bigger than those of ADMPC, which presented stronger intramolecular HBs [59]. In this regard, it is worth mentioning that, in accordance with the experimental data, the CDMPC models constructed over time were found to remain in an elongated conformation, whereas ADMPC formed a more compact structure. Coherently, in more recent studies, the root-mean-square deviation (RMSD) values, determined for enantiomers on the selector surface over 10 ns of MD, profiled fluctuations slightly higher for CDMPC (**mod. 16**) compared to ADMPC (**mod. 15**) [43].

In Franses’s models, the polymer backbone atoms were fixed to their positions during all the simulations because they had no substantial mobility, as deduced from ^13^C cross-polarization magic angle spinning (CP/MAS) and MAS solid state NMR experiments [59]. Using a consistent valence force field (CVFF), the energies of the polymer rods were minimized using MD simulations at 500K for 1 ns, with a time step of 1 fs, using an NVT ensemble, and then again at 298K for 1 ns.

**Figure 9:**
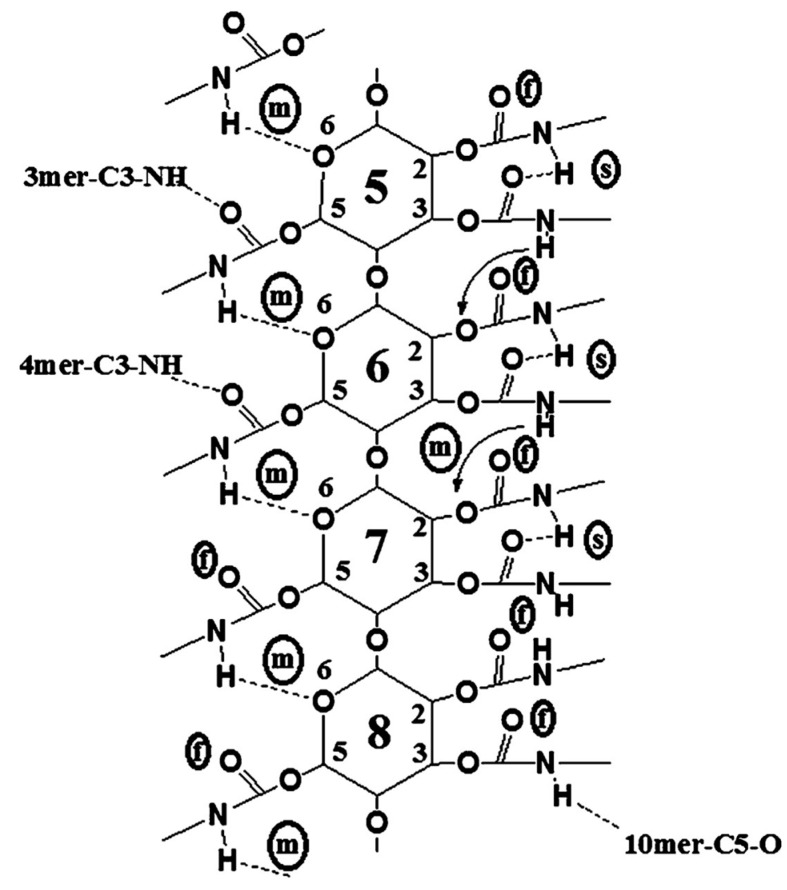
A two-dimensional representation of possible binding sites, NH, CO, and O, of a 12-mer ASMBC polymer model in the central units (monomers 5–8), as predicted from MD simulations (s = strong HB; m = medium-strength HB; f = free (or weakly bonded) group) (Reproduced with permission from ref. [61]. Copyright 2011, American Chemical Society).

Franses’s group also used these models developed with MD in further studies [50,60,61,62]. In particular, the accuracy of CVFF as a force field was evaluated in comparison to density functional theory (DFT) predictions for five types of HBs and one π–π interaction by using the ASMBC model [61]. The CVFF force field used a Morse potential for modeling bond stretching, Coulomb’s law for electrostatic interactions, and a Lennard-Jones function for vdW interactions. HBs were modeled as a combination of electrostatic and vdW interactions. With these features, the models could lead to accurate predictions of the molecular structures but less accurate predictions of the HB energies. The comparison showed that the percentage differences between the CVFF and DFT predictions varied from 7% to 130% for energies, from 1% to 10% for distance, and from 1% to 7% for angles. These differences were significantly smaller than those determined with other force fields like PCFF, COMPASS, Dreiding, and Universal. Furthermore, focusing on the central section of the 12-mer, containing monomers 5–8, to minimize possible chain end effects and considering data averaged by randomly choosing 40 frames from the equilibrium states, details about the binding states of the N-H and C=O groups of ASMBC were gained (Figure 9) [61].

Using MD, the authors could identify three types of NH groups which had either (*a*) a strong (s) bond with C=O groups or (*b*) a medium (m) strength bond with O atoms or (*c*) a weak bond or “free” bond (f), as evaluated from the bond distances and angles. The relative populations of these groups in the MD model were 4:5:3 (f:m:s). However, the percentage of “free” NH groups (about 30%) were slightly larger than the results (about 17%) derived from the IR of the actual polymer and DFT. This suggested the presence of some additional HBs between adjacent molecules in the actual polymer material and the formation of interpolymer HBs. Moreover, simulations of the 12-mer left-handed ASMBC (**mod. 10**) and energy components by mixing the polymer with 200 *n*-hexane molecules were also performed by the same group [50], showing that *n*-hexane did not change the HB state of the polymer but only induced a slight energy-related relaxation of the phenyl groups located in the pendant groups.

In 2010, in accordance with the ADMPC structure defined by Okamoto’s group, Zhou et al. constructed a 36-mer molecular model of ADMPC with a left-hand 4/3 helix (**mod. 9**) [49]. The authors modeled not only the selector but also the overall chiral stationary phase based on the following workflow: (*a*) the γ-aminopropyl silanized silica gel surface was constructed based on the structure proposed by Zhuravlev et al. [63]; (*b*) the two components, the ADMPC and the surface, were pre-optimized to avoid unwanted close contact before combining with each other; (*c*) the side chains of ADMPC and the aminopropyl silane portion of the modified silica gel surface were separately relaxed by an MD simulation, whereas the other parts in the models were kept fixed; (*d*) after the relaxation, the constraint in ADMPC was removed, and the two components were merged into the CSP model. The ADMPC was settled parallel to the silica gel surface at 10.0 Å; (*e*) finally, a two-stage MD simulation was employed for relaxing the model three times, which contained stage I at 1000 K for 0.5 ns and stage II at 273 K for 1.0 ns. Then, an MD equilibration at 273 K was performed for 3.0 ns. The snapshots were collected at an interval of 2500 steps, and the snapshots for the last 1.0 ns were sampled to determine the ADMPC model; (*f*) based on the 36-mer structure, a 13-mer segment was then extracted for carrying out the MD of the selector–analyte interaction. 

In 2016, Huang et al. constructed a 12-mer model of CMB (**mod. 11**) by using the Polymer Consistent Force Field (PCFF) derived from CFF91 [51]. The model was built based on the optimized glucopyranosyl monomer of CMB with the terminal hydroxyl groups replaced by methoxyl groups. In MD simulations, the 12-mer CMB was subjected to 100 ps equilibration at 500 K followed by another 100 ps production at 298 K. Given that conformations were saved every 10 ps, 10 frames were generated from the production, and each single conformation was subsequently minimized. In the frame of this workflow, the medium was treated implicitly, with the dielectric constant set to 1.00.

In 2018, Altomare et al. prepared a 12-mer ADMPC (**mod. 12**) [52] starting from the two left-handed double helix of α-amylose deposited in the PolySac3Db (https://polysac3db.cermav.cnrs.fr/home.html, accessed on 26 October 2023), replicating the structural data achieved by Imberty et al. [64]. Thus, twelve units of one chain were selected, and 3,5-dimethylphenyl-carbamoyl moieties were linked to all the hydroxyl groups of each α-glucose monomer, further relaxing the whole structure with the Maestro software (Release 2016-3) package. To achieve a plausible low-energy conformation representation, the 12-mer model was then subjected to a short MD simulation. Using the Desmond system builder tool implemented in Maestro, a solvated model was assembled, merging the 12-mer in an orthorhombic box filled with MeOH molecules and mimicking the mobile phase used in the chromatographic enantioseparation. All simulations were performed at constant temperature (300 K) and pressure (1 bar) for a total of 180 ns, using the default settings and relaxation protocol of Desmond, with an energy and trajectory recording interval of 60 ps. From the achieved trajectories, the frame featuring the lowest potential energy was selected for further MD studies.

**Figure 10:**
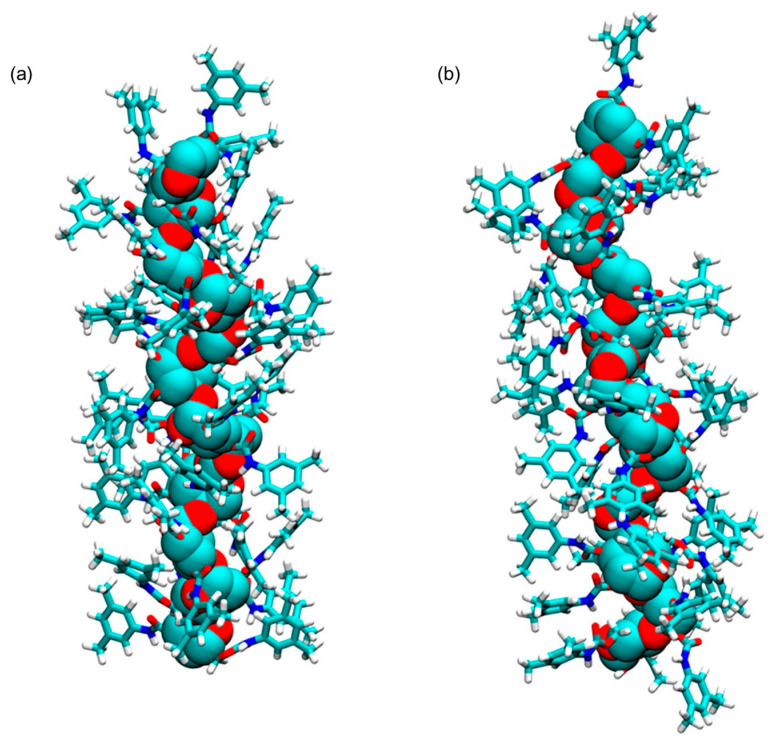
Average structures of ADMPC in MeOH (**a**) and 90/10 *n*-heptane/2-PrOH (**b**). The backbone atoms are represented with VdW spheres, and the pendant groups are represented with sticks. Hydrogen atoms are in white, carbon atoms are in cyan, nitrogen atoms are in blue, and oxygen atoms are in red. (Reproduced with permission from ref. [53]. Copyright 2017, American Chemical Society).

In 2017, Murad et al. prepared the ADMPC as a 12-mer model (**mod. 13**) [53]. In this study, the 12-mer model of the ADMPC reported by Okamoto et al. [29] was used as starting structure to generate the coordinates for the ADMPC monomer for estimating the partial charges. For this purpose, Gaussian 09 [65] was used with the B3LYP/6-31G(d) functional/basis set combination at a DFT level. Then, by using AMBER 14, the 12-mer ADMPC chain was placed into the solvent box to generate the average structure that had the largest population over 100 ns MD simulation, using clustering analysis from the last 20 ns of the simulation. Using MD, the authors evaluated the impact of two explicit solvents on the ADMPC virtual structure, namely methanol (MeOH) and the *n*-heptane/2-PrOH 90:10 mixture. For this purpose, 4826 MeOH molecules and 1159 *n*-heptane/215 propan-2-ol (2-PrOH) molecules were used to mimic the exact concentrations of the experimental conditions based on the density of the solution, the molar mass of the solvent molecules, the composition of the mixture, and the size of the simulation box. As a result, the average virtual ADMPC structure featured the same 4/3 left-handed helical structure with both virtual media. However, for the ADMPC polymer, a more extended average structure by 4.6 Å was observed in *n*-heptane/2-PrOH 90:10 compared to MeOH (Figure 10). The authors ascribed this result to the differences in the distribution of solvent molecules close to the backbone of ADMPC, producing changes in the distribution of the dihedral angles (ϕ, φ) of the glycosidic linkage between adjacent monomers that defined the structure of the polymer according to the values determined by Okamoto et al. [29]. Later, Murad’s group studied **mod. 13** by also using acetonitrile as an explicit solvent [66].

**Figure 11:**
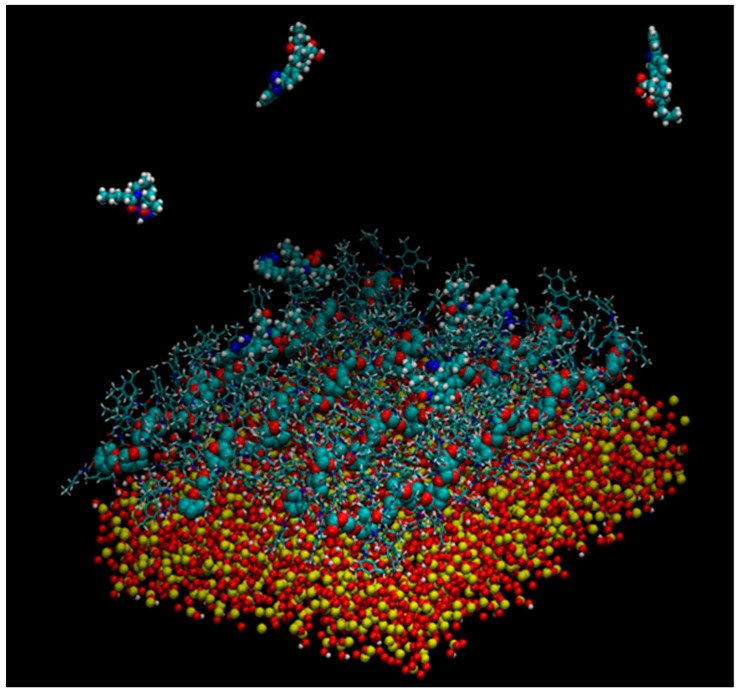
A snapshot of the simulation system multistrand 18-mer ADMPC system developed by Murad et al. [54]: the amorphous silica slab is at the bottom of the simulation box, four 18-mer strands of ADMPC are held on the silanol-capped amorphous silica by van der Waals interactions. Enantiomers of benzoin are also depicted at the interface and in the bulk solvent. For clarity, solvent molecules are not displayed here (Reproduced with permission from ref. [54]. Copyright 2020, American Chemical Society).

Murad’s group also constructed a model of multiple 18-mer ADMPC polymer strands coated on an amorphous silica slab with the dimensions 72.475 × 72.475 × 15 Å^3^ (Figure 11) (**mod. 14**) [54]. The initial silica slab structure was again prepared based on the model proposed by Zhuravlev et al. [63]. Then, the structure was submerged into a 72.475 × 72.475 × 45 Å^3^ box, where it was surrounded by water molecules. By using such a “sandwich” arrangement, the authors intended to increase the contact surface area, facilitating equilibration. The silica slab was equilibrated under MD conditions for 580 ps. Then, four 18-mer ADMPC strands, previously equilibrated for 100 ns in the solvent system (*n*-heptane/2-PrOH or MeOH), were used by the authors to coat the silica slab, and the overall system, after several steps of equilibration, was finally equilibrated for 40 ns. More recently, a 20-mer ADMPC-based virtual CSP was prepared following the workflow developed for **mod. 14** [67], treating the solvent explicitly.

In the last few years, Peluso et al. modeled ADMPC (**mod. 15**), CDMPC (**mod. 16**) [43,55], and CMB (**mod. 17**) [56] by MD, including a solvent explicitly based on the following workflow: (*a*) the Gaussian 09 program (DFT, B3LYP, 3–21G*) was used for the geometry optimization calculation of a monomeric unit of α- and β-D-glucose-1,4-dimethoxy-*tris*(3,5-dimethylphenylcarbamate). The optimized structures were used to build 9-mer of ADMPC and CDMPC, which resulted in agreement with the structure previously reported by Okamoto et al. [28,29]; (*b*) the CMB oligomer was prepared by changing 3,5-dimethylphenylcarbamate, featuring the CDMPC, to 4-methylbenzoate as a pendant group; (*c*) the terminal residues of the biopolymers were closed with methoxyl groups; (*d*) the polymer structures were energy minimized using GAFF force-fields with AM1-BCC charges assigned with the Antechamber toolkit; (*e*) the atoms of the terminal methoxyls, closing the polymer backbone, were fixed in their positions during the simulations by assigning a force constant of 200 kcal/mol so that, starting from the initial values, the backbone dihedral angles of residues 2–8 could moderately rotate on the basis of the applied restriction; (*f*) the structures of the polymer were minimized using 2 ns of MD simulations, using AMBER 18 software; (*g*) *n*-hexane, *n*-hexane/2-PrOH 95:5, or MeOH as solvents were taken into account by means of the explicit periodic solvent box; and (*h*) the production time was 10 ns [43,55], extended to 100 ns in more recent studies [56].

**Figure 12:**
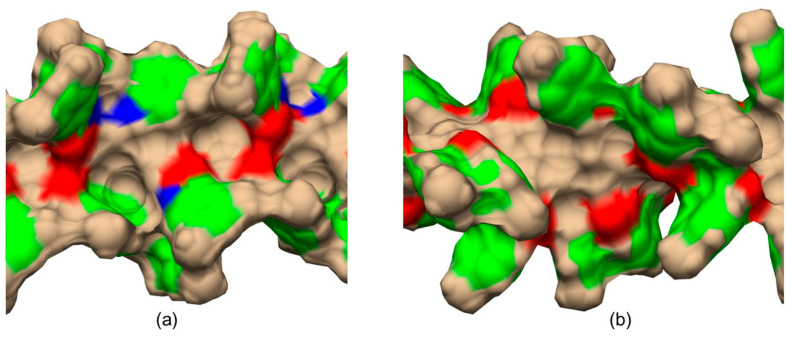
Graphic representations of the shape of cellulose *tris*(3,5-dimethylphenylcarbamate) (CDMPC) (**a**) and cellulose *tris*(4-methylbenzoate) (CMB) (**b**) chiral cavities as derived from 100 ns MD simulations. Color legend: blue, nitrogen; red, carbonyl oxygen; green, phenyl; tan, all other atoms (Adapted with permission from ref. [56]. Copyright 2023, Wiley).

The main difference between CDMPC (Figure 12a) and CMB (Figure 12b) was the absence of the amidic hydrogen atoms (Figure 12a, blue regions) in the benzoate-based selector. 

Very recently, by comparing CDMPC and CMB, how this feature has consequences on the stereoelectronic properties of the chiral cavities featuring the chiral selectors was evidenced [56]. Indeed, given that the pendant groups of the CMB exclusively contained carbonyl oxygen atoms as HB acceptors, intramolecular HBs stabilizing the highly ordered structure of the polymer were thus not possible in the benzoate selector due to a lack of amidic hydrogen atoms as an HB donor counterpart. As a result, the lower stability of CMB compared to the phenylcarbamate derivative has been reported, and, consequently, the chiral recognition properties of CMB proved to be more influenced by the conditions used for the preparation of the packing material [68,69,70]. A comparison between the results of 100 ns MD simulations carried out with 9-mer models representing CDMPC and CMB (Figure 11) showed that the benzoate-based polymer presents slightly smaller cavities than CDMPC, although they are more flexible for conformational adjustment due to the absence of intramolecular HBs.

3,5-Disubstituted phenylcarbamates as pendant groups have been proven to lend the corresponding chiral columns higher versatility toward several classes of chiral compounds, irrespective of substituent type. As a possible explanation of this behavior, it was hypothesized that the substituent position on the carbamate phenyl ring could contribute to determine the shape of the chiral cavities hosting the chiral analyte. Very recently, to confirm this hypothesis and to determine the origin of the higher performances of polysaccharide *tris*(3,5-disubstituted phenylcarbamates) at a molecular level, three amylose 4/3 left-handed helical phenylcarbamate-based 9-mer featuring (*a*) two methyl groups at the positions 3 and 5 (ADMPC), (*b*) one methyl group at the position 4, and (*c*) two methyl groups at the positions 2 and 5 of the phenylcarbamate pendant groups, respectively, were built as virtual polymers, and the shape of their chiral cavities was explored with MD, carrying out 100 ns MD simulations, with the mixture *n*-hexane/2-PrOH 90:10 as an explicit virtual solvent [32]. 

**Figure 13:**
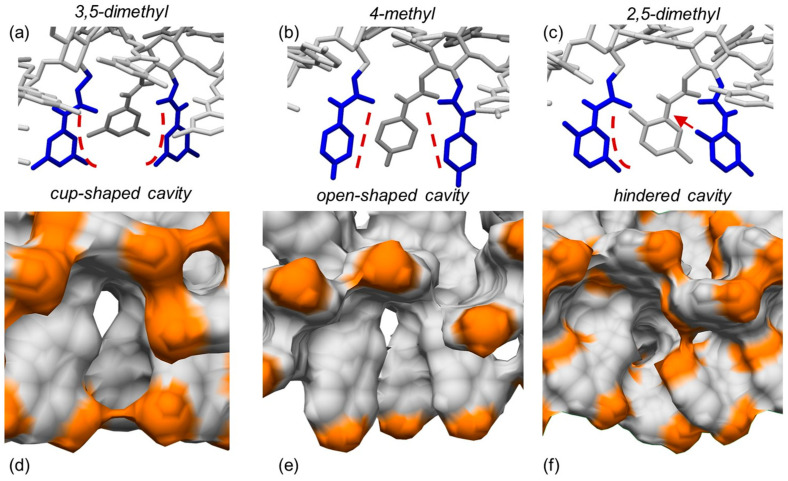
Typical shapes of the chiral cavities into amylose carbamate-based chiral stationary phases modeled as drawing structures (**a**–**c**) and as electron density surfaces ((**d**–**f**), orange, methyl groups): 3,5-dimethylphenylcarbamate (**a**,**d**), 4-methylphenylcarbamate (**b**,**e**), 2,5-dimethylphenylcarbamate (**c**,**f**) (Reprinted with permission from ref. [32]. Copyright 2023, Elsevier).

The results of the simulations carried out in this study supported the initial hypothesis, and three different cavity shapes corresponding to the three different substitution patterns emerged from the MD simulations (Figure 13): (*a*) cup-shaped cavities for the 3,5-disubstitution (a), (*b*) open-shaped cavities for the 4-substitution (b), and (*c*) cavities hindered by the 2-methyl protruding inside the groove in the case of the 2,5-disubstitution (c). Based on these results, the “open-shaped cavity” and the “hindered cavity” could be too large for small analytes and too small for large-sized analytes, respectively. On the contrary, the “cup-shaped cavity” appeared to be geometrically more adaptable toward analytes of a different size. These results provided a rational explanation at a molecular level for the higher versatility of polysaccharide *tris*(3,5-disubstitutedphenylcarbamates) as chiral selectors in enantioseparation science.

## 5. Application of MD to Model Enantioseparations Promoted by Polysaccharide-Based Selectors

Over time, MD simulations to model enantioseparations promoted by polysaccharide-based selectors were reported for alkylated amylose and cellulose derivatives like ADMPC, ASMBC, CDMPC, and CMB (Table 2). 

On the contrary, models optimized with MD for chlorinated polysaccharide-based selectors are practically missing except for a recent model reported for cellulose *tris*(3-chloro-4-methylphenylcarbamate) (CCMPC) by Aboul-Enein et al. [74]. Unfortunately, in this study, details about the construction of the initial structure of the virtual polymer were missing.

**Figure 14:**
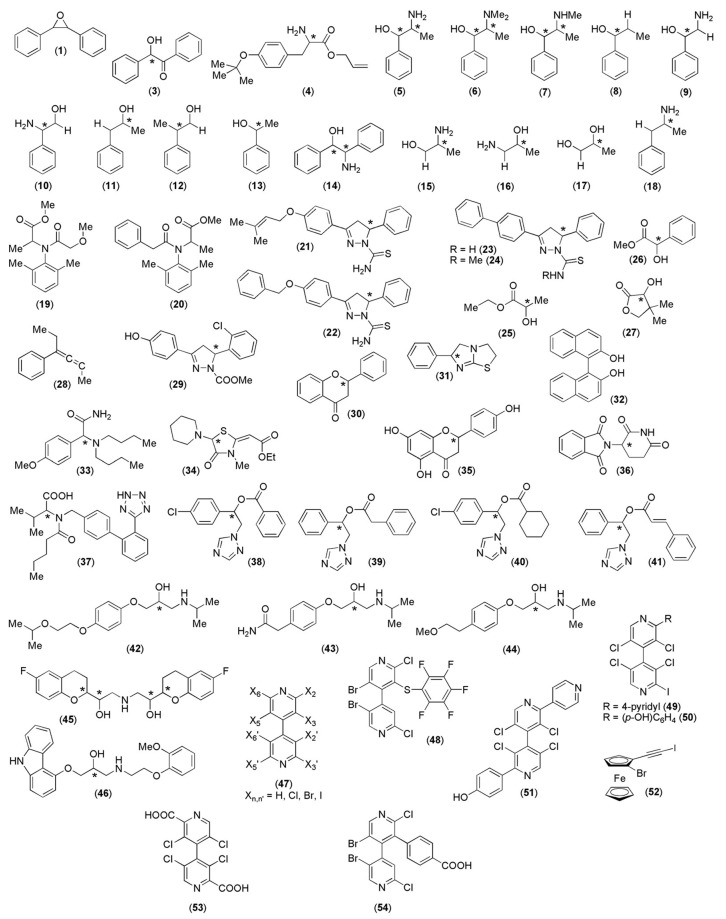
Chiral analytes enantioseparated on polysaccharide-based selectors and modeled as polysaccharide-based complexes with MD. Stereogenic centers are indicated with an *.

Although the first attempts to use MD to model polysaccharide derivatives date back to the 1990s, and complexes of more than fifty analytes (Figure 14) were modeled so far using MD, methods of general applicability for polysaccharide-based selectors and related enantioseparation processes are still missing. In most cases, available models were developed on a case-by-case basis, as highlighted by the variety of force fields, software, conditions, and boundary conditions (Table 1 and Table 2) used so far.

Most modeled analytes feature central chirality (77%), whereas only 21% of the analytes contain a chiral axis as a stereogenic element, and only a single case of an MD simulation of a planar chiral ferrocene (analyte **52**) was reported so far.

The first successful approach based on integration of experimental analysis and molecular modeling was reported in 2007 by Wirth et al. [48] to explore the origin of the unusually high enantioselectivity (α = 16) observed for the *p*-*O*-*tert*-butyltyrosine (**4**) with an ADMPC-based CSP and chloroform as mobile phase. Moreover, acidity proved to switch on the enantioselectivity, increasing the retention time of the L-enantiomer only, while the retention of the D-enantiomer was changed only slightly. In order to understand the origin of this evidence, an NMR spectroscopy was used, and 2 ns MD was carried out by exploiting a 12-mer ADMPC (**mod. 5**) and implicit chloroform. Based on the MD data, in agreement with the results of the NMR experiments, for the protonated enantiomers, the van der Waals interactions contributed almost like electrostatic interactions to the enantioselectivity, and both contributions were about 3.5 kcal·mol^−1^ more favorable for the L-enantiomer compared to the D-enantiomer (enantiomer elution order (EEO) = D-L). To understand why acid switched on the selectivity (EEO = L-D) rather than augmenting the enantioselectivity, the role of the acid was studied computationally by removing the proton from the analyte and performing MD simulations for these deprotonated enantiomers. The electrostatic contribution was practically nullified for both enantiomers via deprotonation. The van der Waals interactions changed little for the L-enantiomer, but they became significantly more favorable for the D-enantiomer. The total binding energy became almost identical for the two enantiomers upon deprotonation, which agrees with the low chromatographic selectivity observed without acid. 

In 2008, Franses’s group studied the key interactions of norephedrine (**5**) with ADMPC, ASMBC, and CDMPC by using ATR-IR and molecular simulations [22]. Given (+)-(−) as the EEO, observed for **5** under chromatographic conditions, the MD simulations (1 ns) of the polymer–**5** binary systems (solvent was neglected in this modeling) were consistent with the chromatography results obtained with *n*-hexane/2-PrOH 90:10 as a mobile phase: (*a*) a higher enantioselectivity for the ADMPC, (*b*) no enantioselectivity for the ASMBC, and (*c*) a low selectivity value for the CDMPC. Using MD, the significant enantioselectivity observed in the ADMPC (**mod. 6**) was ascribed to three simultaneous interactions (two HBs and one π–π) of the polymer with (−)-(**5**) versus two interactions (one HB and one π–π) with (+)-(**5**). In the same years, the authors explored the impact of the molecular structures of chiral analytes **5–17** on their high-performance liquid chromatography (HPLC) retention and selectivity on the CDMPC. Among these analytes, only **6** showed significant selectivity [58]. The HB interactions between the functional groups of the analyte and the C=O and N-H functional groups of the polysaccharide were probed with ATR-IR. As a result, the CDMPC IR amide band wavenumbers changed significantly, indicating HB interactions between the carbamate moiety of the polymer and the chiral analyte. The EEOs predicted for the enantiomers of the chiral solutes using MD of the CDMPC (**mod. 8**)-analyte binary systems agreed with the HPLC results. The MD results were consistent with the three-point attachment model, profiling a combination of steric hindrance, HB, and π–π interactions as molecular bases for the enantioseparation. Later, similar results were obtained by applying the same approach to explore the molecular bases of the enantioseparation of chiral analytes **5**–**18** with the ADMPC [60]. 

In 2010, Zhou et al. studied the mechanism of the enantioseparation of metalaxyl (**19**) and benalaxyl (**20**) with ADMPC (**mod. 9**) using MD [49]. EEOs and binding energies derived from calculations agreed with the experimental results. The chiral separations of **19** and **20** were mainly affected by HBs, although the π–π and NH–π interactions were also found to play a role in enantiorecognition. Although the virtual CSP consisted of the 36-mer ADMPC-settled parallel to a modeled silica gel surface located at 10.0 Å, the silica gel surface contributed very little to the separations in terms of direct interactions with enantiomers. On this basis, the authors argued that, although the surface appeared to be not involved in the composition of the chiral cavity hosting the analyte, it contributed to keep the ADMPC fixed on the surface, with a stable conformation, avoiding an arbitrary deformation of the side chain. In this regard, it is worth mentioning that deformation of the polymer can be avoided by applying energy constraints to the polysaccharide backbone and side chains during MD production (for instance, see the discussion of the next application).

**Figure 15:**
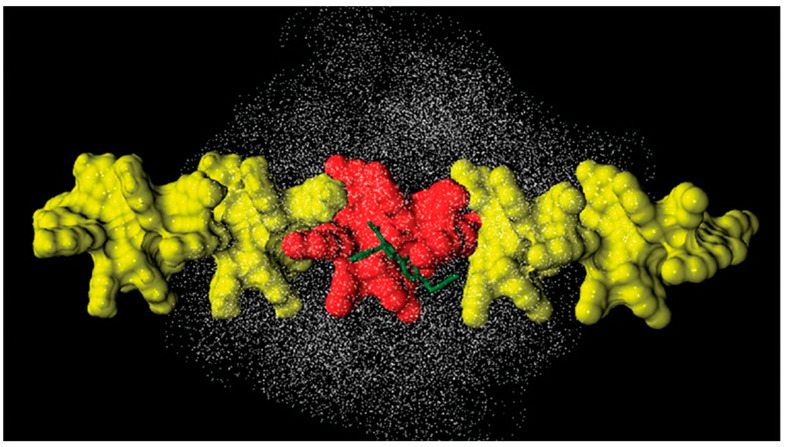
Scheme of an MD model of a CMB-promoted enantioseparation of compounds **21** and **22** (viewpoint is perpendicular to the chain axis): docking selector model (4-mer) of CMB is colored in red, units added in the MD model (16-mer) are colored in yellow, analyte is in green (Reproduced with permission from ref. [71]. Copyright 2012, American Chemical Society).

In 2012, Alcaro, Cirilli et al. modeled the enantioseparation of chiral pyrazole derivatives **21** and **22** on a CMB-based selector to explore the recognition mechanism underlying these enantioseparations [71]. By using ethanol (EtOH) as an MP, a very high enantioselectivity was reported for both **21** (α = 73.2) and **22** (α = 79.8), with (*R*)–(*S*) as EEO in both cases. In this study, the polysaccharide-based selector was modelled starting from the left-handed threefold 3/2 helix CTPC model described by Okamoto’s group [28], replacing the carbamate with a benzoate moiety. A combined theoretical approach was used to explore the nature of the selector–enantiomers association, which consisted of (*a*) docking analysis by using a 4-mer oligomer as a virtual selector (Figure 15, red units), and (*b*) MD simulation (60 ns, EtOH as explicit solvent) by using a 16-mer oligomer (Figure 15, red 4-mer + 12 yellow units). To prevent unrealistic distortions of the CMB model, the glucopyranosyl ring atoms were restrained in their position by means of a constant force equal to 200 kcal/mol. Based on this protocol, the chiral recognition of **21** and **22** on the CMB was observed to be driven by hydrophobic and π–π noncovalent interactions rather than by HB contributions. Indeed, the thioamide moiety, the pyrazole nitrogen, and the ether oxygen were mainly found to establish HBs with the explicit solvent.

Later, the same group reported a study integrating chromatographic analyses, thermodynamic Van’t Hoff analyses, and MD to explore the recognition mechanism associated with the exceptional enantioseparation of pyrazole derivative **23** (α = 207) with respect to the analogue compound **24** (α = 38) on a CMB-based CSP under NP elution conditions (*n*-hexane/2-PrOH 30:70) [72]. In all cases, (*R*)–(*S*) were determined as EEO. From a comparison of the experimental chromatographic data of **23** and of its *N*-methyl analogue **24** with those obtained with MD, selectivity was found to be modulated by HB interactions established between the thioamide moiety of the analyte and the carbonyl oxygen atoms of the selector. Each variation driven to weaken HBs caused a decrease in enantioselectivity. MD simulations also showed that, for both analytes, the common biphenyl moiety was located between two 4-methylbenzoate pendant groups through vdW and π–π noncovalent interactions, whereas HBs were observed between the -NH thioamide group and the carbonyl oxygen atoms of the selector. Nevertheless, due to the methyl thioamide, the (*S*)-enantiomer of **24** could interact with the selector through only one HB, while for (*S*)-**23**, two HBs were observed.

Between 2011 and 2013, Franses et al. applied the approach previously developed, again based on the integration of IR analysis and MD simulation, to study mechanisms and noncovalent interaction patterns underlying the enantioseparation of analytes **3** and **25–27** with ASMBC, which was modeled as 12-mer **mod. 7** [61] or **mod. 10** [50].

In 2014, Grinberg et al. reported the enantiomeric separation of the aromatic allene **28** using a commercially available CSP based on CDMPC [62]. In this study, molecular VCD spectroscopy, DFT calculations, MM, and MD were used to simulate the interaction of each enantiomer with the CSP. For this purpose, from the DFT/IR/VCD results, initial binding configurations for further simulations were obtained, whereas from MD results, the most likely binding cavities and binding sites of the CDMPC could be identified. The modeled structures of the two enantiomers of **28** on the CDMPC (**mod. 8**) surface showed the (*R*)-enantiomer confined into the cavity of the selector, with an electrostatic interaction occurring between the cumulene moiety linked to the phenyl ring of the analyte and the carbonyl of the carbamate functional group of the polymer. Otherwise, the (*S*)-enantiomer exhibited strong steric hindrance due to the methyl group of allene, which moved the enantiomer slightly out of the cavity, weakening (*S*)-enantiomer–polymer contact.

In 2016, Huang et al. modeled the enantioseparation of the chiral pyrazole derivative **29** with a 12-mer CMB (**mod. 11**) by means of 100 ps MD, considering seven solvents or mixtures as implicit media [51]. For this purpose, dielectric constant (DC) values were set by the authors to represent the experimental conditions as follows: *n*-hexane/EtOH 70:30 (DC = 9.06), *n*-hexane/2-PrOH 60:40 (DC = 8.58), pure EtOH (DC = 25.80), and pure 2-PrOH (DC = 18.62). In addition, three benchmark solvent conditions, vacuum (DC = 1), pure *n*-hexane (DC = 1.89), and water (DC = 81.00), were also considered to systematically investigate the solvent effect. In polar solvents, the (*S*)-enantiomer/CMB complex appeared more stable than the (*R*)-enantiomer/CMB complex, in accordance with the experimental EEO reported for **29**. Using 2-PrOH as an implicit solvent, while the (*R*)-enantiomer appeared uninvolved in specific interactions, the (*S*)-enantiomer protruded into the groove, which was surrounded by the side chains of the selector. In this case, π–π interactions were formed between the analyte and selector. Using MD, the authors observed the formation of HB between the analyte and CMB in vacuum (DC = 1.00) and in *n*-hexane (DC = 1.89), exclusively. These observations were also confirmed by calculating *E*_vdW_ energies lower than the *E*_el_ contribution to energy interactions, with the interaction energy of the (*R*)-enantiomer/CMB complex higher than the energy of the (*S*)-enantiomer/CMB complex. Thus, calculations predicted an inversion of EEO [(*S*)-(*R*)] in a non-polar medium and in the vacuum where HBs could occur between the hydroxyl proton of **29** and the carbonyl oxygen of the CMB. Nevertheless, experimental confirmation for this prediction was not reported, affecting the reliability of the conclusions reported.

In 2017, 100 ns MD simulations were performed by Murad et al. to elucidate the chiral recognition mechanism of the enantioseparation of flavanone (**30**) enantiomers on a 12-mer ADMPC (**mod. 13**) [53], which were constructed based on the parametrization reported by Okamoto’s group [29]. MeOH or *n*-heptane/2-PrOH (90/10) were used as explicit solvents. In this study, it was found that the number of solvent molecules around each enantiomer of **30** correlated well with the interaction between the ADMPC and the enantiomer in terms of electrostatic interaction energy. Indeed, the number of solvent molecules within 5 Å of the drug molecule significantly decreased when the drug molecule interacted with the ADMPC polymer. In the absence of the ADMPC, the enantiomers formed HBs with MeOH or 2-PrOH, or hydrophobic interactions with *n*-heptane, generating a stable first solvation shell. MD analysis also showed that the lifetime of the HBs formed between analyte enantiomers and ADMPC polymer correlated well with experimental EEO [(*R*)-(*S*)] and the (*S*)-flavanone had a longer HB lifetime with ADMPC than the (*R*)-enantiomer. On the contrary, π–π noncovalent interactions did not impact the separation factor, whereas steric effects between analyte and selector did play an important role in tuning selector–analyte HBs, which were the main noncovalent interactions underlying the enantioseparation. Later, the same group extended this approach to the modeling of analytes **1**, **3**, and **31**–**37** [66], considering *n*-heptane/2-PrOH, MeOH, 2-PrOH, and acetonitrile as explicit solvents. In these cases, positive co-operation between π–π interactions and HBs was observed to be an important factor for enantiorecognition.

In 2020, by using a model that mimicked the real CSP system through a multistrand 18-mer ADMPC system coated on an amorphous silica slab (**mod. 14**), Murad’s group investigated the enantioseparation of **3**, **30**, **36**, and **37** in a 200 ns MD [54]. MeOH, or a *n*-heptane/2-PrOH 90:10 mixture, was used as explicit media. The authors observed that the proposed model provided the possibility for the enantiomer to simultaneously interact with two polymer strands, observing such contact for analyte **37** with *n*-heptane/2-PrOH 90:10 as the explicit medium. On the other hand, for **3** and **30**, MD results that were obtained with **mod. 14** were not dissimilar compared to those obtained with the single strand model (**mod. 13**). On the contrary, the authors stated that **mod. 14** provided a more accurate description of the enantioselective recognition of **36** and **37** in terms of the lifetimes of the HBs underlying analyte–selector contact, providing results in agreement with the experimental EEO. Later, the authors also applied the same MD approach for the study of the enantioseparation of analytes **1**, **31**, and **35** by using ADMPC (**mod. 14**) as a virtual CSP [73]. 

By using a modified version of **mod. 14** containing 20-mer ADMPC strands and a diverse combination of force field/software (GROMOS54A7/LAMMPS [74]), Ciriaco et al. explored the molecular mechanisms underlying the enantioseparation of chiral compounds **38**–**41** in a 100 ns MD carried out in the vacuum, and with MeOH or *n*-hexane/MeOH 90:10 as explicit solvents, in accordance with chromatographic analysis [67]. The authors found consistent differences between each enantiomer throughout the trajectories, revealing that HB, steric hindrance, and π–π interactions played major roles in enantiomeric separation. The HBs formed between the carboxyl oxygen atoms of the analyte and the amidic hydrogen atoms of the ADMPC were the main interactions driving binding and enantioselective recognition. Depending on the positioning of the enantiomers in the chiral groove of the polymer, π–π interactions between the triazole ring and the phenyl rings of the ADMPC pendant groups also had a significant effect on analyte–selector contact.

Very recently, Aboul-Enein et al. carried out 500 ps MD simulations to investigate the solvent effect on the interaction of the chiral β-adrenergic blockers **42**–**46** with CCMPC [74], considering EtOH as the explicit solvent. However, the MD study presents several flaws, in particular, (*a*) details on the construction of the virtual polymers are missing, and (*b*) the virtual solvent (EtOH) included in the MD system was not consistent with the mobile phases (*n*-hexane-based mixtures) used experimentally.

**Figure 16:**
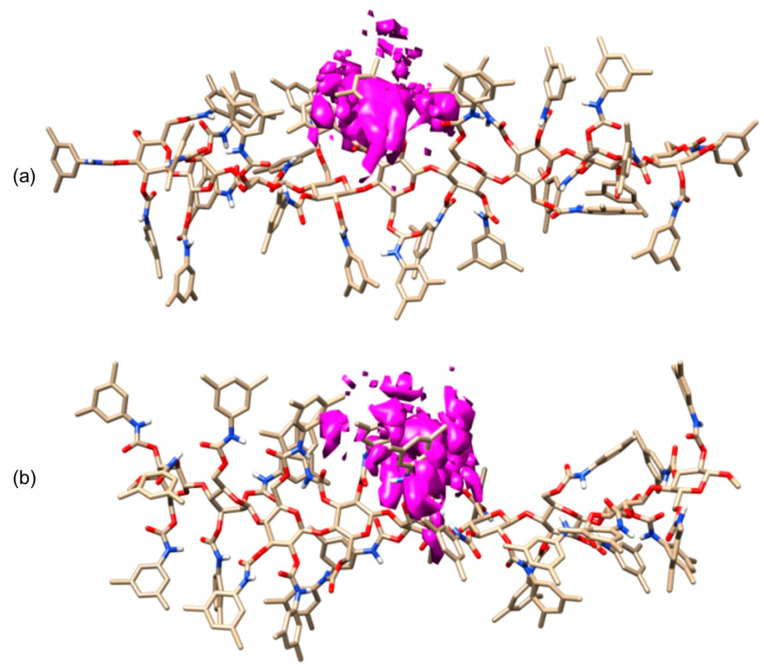
MD simulations of CDMPC/**47** (X_n,n′_ = I) complexes (10 ns), a comparison of occupancy graphs: CDMPC/(*M*)-**47** (X_n,n′_ = I) (**a**), CDMPC/(*P*)-**47** (X_n,n′_ = I) (**b**) (Reproduced with permission from ref. [43]. Copyright 2018, Elsevier).

In 2018, in the frame of a study on halogen bond (HaB)-driven enantioseparations, the interaction modes of eight polyhalogenated 4,4′-bipyridines (**47**) with 9-mer ADMPC (**mod. 15**) and CDMPC (**mod. 16**) were simulated by 10 ns MD [43], using *n*-hexane or MeOH as explicit solvents. A total of 35 X···O and 10 X···π (X = Cl, Br, I) contacts were found, with a clear prevalence of I···O contacts (32). In Figure 16, the results of 10 ns MD production are reported for the enantioseparation of compound **47**, with X_n,n’_ = I, in terms of occupancy graphs evidencing the room occupied by each enantiomer during the production time. Based on this picture, the (*P*)-enantiomer penetrated more deeply into the groove in accordance with the sequence (*M*)–(*P*) observed as the experimental EEO. Moreover, the involvement of the carbonyl sites as HaB acceptors (nucleophiles) was statistically evaluated, and the highest values were observed for the carbonyl at 3- and 6-positions of the glucopyranosyl residues [55]. Interestingly, the number of interactions observed for the iodinated, brominated, and chlorinated compounds **47** tended to reflect the order I > Br > Cl, in agreement with the σ-hole depth trend and experimental outcomes. In these studies, the explicit σ-hole (ESH) treatment was used to model the σ-hole region on halogen atoms, accounting for their electrophilic properties as HaB donors [43]. For this purpose, a massless dummy atom connected to I, Cl, and Br was introduced manually by using 1, 1.3, and 1.6 Å as distance and 0.1, 0.2, and 0.3 units of positive charge for the extra point for Cl, Br, and I, respectively. To keep the total charge of the molecule unchanged, an equivalent negative charge was manually added to each halogen atom.

Later, the same strategy was applied to the modeling of the fluorinated sulfur-containing 4,4′-bipyridine **48** with CDMPC (**mod. 16**), with the aim of detecting and studying the chalcogen bond (ChB) in solution [78]. The possibility of ChB-driven enantioseparation was confirmed via chromatographic analysis and MD. 

In 2021, our group carried out a series of 100 ns MD under explicit solvent conditions by using 9-mer ADMPC (**mod. 15**) and CDMPC (**mod. 16**) as virtual polymers as well as analytes **49–51** [75,76]. On this basis, how the interplay between HB and HaB can drive enantioselective recognition in a way that is dependent on the polysaccharide backbone was demonstrated. Indeed, as halogen atoms are much larger and polarizable than hydrogen, HaB is more sensitive to steric hindrance than HB. Thus, given that ADMPC showed more compact cavities compared to CDMPC, this latter was more prone to form HaBs with iodinated analytes.

**Figure 17:**
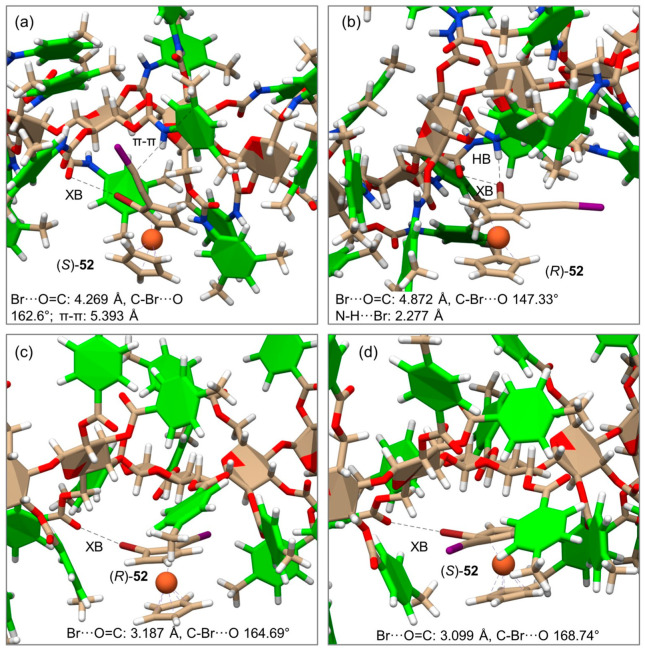
Representative snapshots and noncovalent interactions from the simulated molecular dynamic (MD) trajectories of the complexes of (*R*)- and (*S*)-**52** with CDMPC (**a**,**b**), and CMB (**c**,**d**) (Reproduced with permission from ref. [56]. Copyright 2023, Wiley).

Very recently, the enantioseparation of the halogenated planar chiral ferrocene **52** on CDMPC and CMB was comparatively studied, and MD simulations were performed to unravel the molecular bases of the enantioseparation with both selectors [56]. For this purpose, CDMPC (**mod. 16**) and CMB (**mod. 17**) were used as chiral selectors under explicit solvent conditions (*n*-hexane/2-PrOH 95:5). The MD results disclosed that HaBs can also participate in the recognition mechanism of halogenated ferrocenes on cellulose-based selectors with an efficacy dependent (*a*) on the properties of the selector as an HaB acceptor and (*b*) on the presence of competitive noncovalent interactions that may oppose or weaken HaBs. Thus, ferrocene **52** showed different noncovalent interaction patterns with the two selectors (Figure 17), and for the CMB-complexes, shorter distances and angle values closer to the reference value of 180° indicated the presence of stronger HaBs compared to those observed with the CDMPC.

These simulations represented the first attempt to model the enantioseparation of planar chiral ferrocenes on cellulose-based selectors.

Finally, by using the 9-mer ADMPC (**mod. 15**) in 100 ns MD, treating the *n*-hexane/2-PrOH 90:10 mixture as an explicit solvent, the mechanisms of the enantioseparation of 4,4′-bipyridines **53** and **54** were also studied [77]. In accordance with experimental observations, an MD analysis allowed for reasonable conclusions to be drawn: (*a*) at a molecular level, the high retention of compound **53** could be ascribed to the strong HB ability of the two COOH groups at the 2,2′-positions of the heterocyclic scaffold; (*b*) the limited enantiodiscrimination between the atropisomers of diacid **53** could be due to the symmetry of the HB system, involving the COOH groups at the termini of the molecule and to the 3,3′,5,5′-tetrachloro symmetric pattern around the 4,4′-bond as a chiral axis; (*c*) the enantioselective recognition of the atropisomers of acid **54** could be explained at a molecular level with the occurrence for the most retained (*M*)-enantiomer of an enantioselective π–π interaction between the phenyl ring of the analyte bearing the COOH group and the 3,5-dimethylphenyl functionality of the selector. This interaction was not observed for the (*P*)-**54** complex, because the carboxyphenyl group of the analyte protruded outside the polymer groove.

## 6. Conclusions

MD simulations represent a pivotal tool to model polysaccharide-based selectors and related enantioseparations. This technique, introduced in this field in the 1990s, offers a unique possibility to model real-life liquid phase enantioseparations promoted by polysaccharide-based selectors, accounting for the dynamics of the process, and for solvation effects. However, an examination of the MD studies presented and discussed in this review highlighted that, until now, the methods of general applicability for polysaccharide-based selectors and related enantioseparation processes are still missing, and in most cases, reported models were developed on a case-by-case basis. Indeed, several different software, force fields, and workflows were used, and this may lead to the lack of algorithmic interoperability between codes, resulting in the publication of a series of non-confrontable and non-homogeneous results. Furthermore, different polymer fragments as virtual selectors and boundary conditions were also used. Another critical aspect concerns the fact that crystallographic structures are missing for most polysaccharide-based selectors available in the market. Even though reliable structural information was derived from spectroscopic NMR, IR, and VCD analysis, in some cases, modeling polysaccharide-based systems was approached on heuristic bases that still need proper validation. Most polysaccharide models reported so far were prepared based on the structures developed by Okamoto’s group for CTPC [28] and ADMPC [29]. Two models for the CSP system consisting of polysaccharide combined with a silica slab were reported so far. However, the actual advantages of using these CSP models compared to modeling the polymer alone are still debatable.

The knowledge of the absolute configuration of the enantiomers and their EEO under chromatographic conditions are important benchmark pieces of information to validate the reliability of the model by evaluating the agreement of calculated and experimental EEO. Moreover, different force fields can be fruitfully used and compared to verify the occurrence of abnormal results and structural distortions related to the choice of unsuitable force fields.

Sometimes, approximations were applied to make the study feasible in terms of computation cost. However, these “tricks” must be consistent with boundary conditions, not affecting the reliability of the model and its capability to properly describe the real system. Unsuitable approximations may seriously affect the reliability and the predictive capability of the model. Among possible approximations, neglecting solvent or treating it implicitly should be avoided. The mobile phase is an essential component of the chromatographic system—a powerful tool to modulate noncovalent interactions potentially occurring between analyte and selector. Treating a solvent explicitly, introducing “physically” solvent molecules in the virtual system, and allowing solvent molecules to interact with a modeled selector and analyte enables the model to describe solvent (mobile phase) effects and the energetic components of the system more accurately. Finally, the polymeric nature of real polysaccharide derivatives cannot be neglected. Therefore, these selectors must be modeled in a sufficiently extended form, and simulations of small frameworks of a selector consisting of three or five units should be avoided.

On the one hand, state-of-the-art MD techniques proved to be reliable enough to describe the impact of structural features of analytes and selectors on enantioseparation. The integration of MD and experimental techniques was fruitfully applied to identify σ-hole interactions like HaB and ChB in an HPLC environment. On the other hand, (*a*) the application of MD to model chlorinated polysaccharide-based selectors, (*b*) the capability of MD models to describe the impact of mobile phase changes on enantioseparation, (*c*) the construction of polymer/silica systems able to describe the impact of the immobilization technique (coating or covalent) on the enantioseparation, and (*d*) the application of MD to study the enantioseparation of organometallic chiral compounds, planar chiral compounds, and dispersion forces that drive chromatographic enantioselective recognition remain issues still open in this field. In this regard, further efforts will be needed in the future to improve the reliability and predictive ability of MD-based models to describe polysaccharide derivatives and related liquid phase enantioseparations.

Overall, the main aim of the computational investigations based on the MD of liquid-phase enantioseparations is to develop reliable predictive models to improve the efficiency of experiments and to speed up scientific advancements. In this perspective, it is worth mentioning that machine learning techniques may also be useful to predict the retention times of enantiomers and facilitate chromatographic enantioseparation [79,80]. Although they are developed on different conceptual bases, both MD and machine learning techniques are expected to provide useful predictive models in the future, even if further efforts are needed to improve the reliability of the proposed models and their applicability for a wide range of analytes, selectors, and mobile phases.

## Figures and Tables

**Table 1:** Models of commercially available polysaccharide-based selectors built by MD simulations: polymer acronym, model type, force field/software, solvent, year [Ref.] ^1^.

Polymer	Model (mod. *n*)	Force Field/Software	Production Time	Solvent	Year [Ref.]
CTPC	8-mer (**mod. 1**)	CHARMM/CHARMM	10 ps	Not considered	1995 [28]
CTA	multistrand 8-mers (**mod. 2**)	AMBER/Macromodel	300 ps	Not considered	1997 [46]
CTPC, CDMPC	9-mer (**mod. 3 and 4**)	CHARMM/CHARMM	10 ps	Not considered	1999 [47]
ADMPC	12-mer (**mod. 5**)	COMPASS/MS-Modeling	200 ps	Implicit CHCl_3_	2007 [48]
ADMPC, ASMBC	12-mer (**mod. 6 and 7**)	CVFF/MS-Modeling	1 ns	Not considered	2008 [22]
CDMPC	9-mer (**mod. 8**)	CVFF/MS-Modeling	1 ns	Not considered	2008 [22]
ADMPC	36-mer/silica surface (**mod. 9**)	COMPASS/MS-Modeling	3 ns	Not considered	2010 [49]
ASMBC	12-mer (**mod. 10**)	CVFF/MS-Modeling	3 ns	Explicit Hex	2013 [50]
CMB	12-mer (**mod. 11**)	PCFF/Material Studio	100 ps	Not considered	2016 [51]
ADMPC	12-mer (**mod. 12**)	Not specified/Maestro	180 ns	Explicit MeOH	2018 [52]
ADMPC	12-mer (**mod. 13**)	GAFF/AMBER	100 ns	Explicit Hept/2-PrOH,	2017 [53]
				MeOH	
ADMPC	multistrand 18-mers/silica	GAFF/AMBER	40 ns	Explicit Hept/2-PrOH	2020 [54]
	surface (**mod. 14**)				
ADMPC, CDMPC	9-mer (**mod. 15 and 16**)	GAFF/AMBER	10 ns	Explicit Hex or MeOH	2018 [43,55]
CMB	9-mer (**mod. 17**)	GAFF/AMBER	100 ns	Explicit Hex/2-PrOH	2023 [56]

^1^ Amylose *tris*(3,5-dimethylphenylcarbamate), ADMPC; Amylose *tris*((*S*)-α-methylbenzylcarbamate), ASMBC; Cellulose triacetate, CTA; Cellulose *tris*(4-methylbenzoate), CMB; Cellulose *tris*(3,5-dimethylphenylcarbamate), CDMPC; Cellulose *tris*(phenylcarbamate), CTPC; *n*-Heptane, Hept; *n*-Hexane, Hex; Propan-2-ol, 2-PrOH; Methanol, MeOH.

**Table 2:** MD simulations of enantioseparations promoted by polysaccharide-based selectors (for the numbering of chiral analytes, see Figure 13).

Polymer Model (mod. *n*) ^1^	Chiral Analyte	Force Field/Software	Time	Solvent	NCI	[Ref.]
ADMPC (**mod. 5**)	**4**	COMPASS/MS-Modeling	2 ns	Implicit CHCl_3_	HB, vdW	[48]
ADMPC, ASMBC, CDMPC	**5**	CVFF/MS-Modeling	1 ns	Not considered	HB, π–π	[22]
(**mod. 6–8**)						
CDMPC (**mod. 8**)	**5–17**	CVFF/MS-Modeling	1 ns	Not considered	Steric, HB, π–π	[58]
ADMPC (**mod. 6**)	**5–18**	CVFF/MS-Modeling	1 ns	Not considered	Steric, HB, π–π	[60]
ADMPC (**mod. 9**)	**19, 20**	COMPASS/MS-Modeling	3 ns	Not considered	HB, π–π, NH–π	[49]
ASMBC (**mod. 7)**	**3**	CVFF/MS-Modeling	3 ns	Not considered	HB, π–π	[61]
CMB ^2^	**21, 22**	OLPS_2005/Desmond	60 ns	Explicit EtOH	Hph, π–π	[71]
CMB ^2^	**23, 24**	OLPS_2005/Desmond	60 ns	Explicit EtOH	HB, π–π	[72]
ASMBC (**mod. 10**)	**3**, **25–27**	CVFF/MS-Modeling	300 ps	Not considered	HB, π–π	[50]
CDMPC (**mod. 8**)	**28**	CVFF/MS-Modeling	500 ps	Not considered	El, Rep	[62]
CMB (**mod. 11**)	**29**	PCFF/Material Studio	100 ps	Implicit Hex/ROH,	HB, π–π	[51]
				ROH, water		
ADMPC (**mod. 13**)	**1**, **3**, **30–37**	GAFF/AMBER	100 ns	Explicit Hept/2-PrOH,	Steric, π–π, HB	[53,66]
				MeOH, ACN		
ADMPC (**mod. 14**)	**1**, **3**, **30**, **31**, **35–37**	GAFF/AMBER	200 ns	Explicit Hept/2-PrOH	HB, π–π	[54,73]
ADMPC (modified **mod.14**)	**38–41**	GROMOS54A7/LAMMPS	100 ns	Explicit solvents	Steric, HB, π–π	[67]
CCMPC ^3^	**42–46**	EHT/AMBER	500 ps	Explicit EtOH	Hph, HB, π–π	[74]
ADMPC (**mod. 15**)	**47**, **49–51**, **53**, **54**	GAFF/AMBER	10–100 ns	Explicit solvents	Hph, HaB, HB, π–π	[43,55,75,76,77]
CDMPC (**mod. 16**)	**47–52**	GAFF/AMBER	10–100 ns	Explicit solvents	HaB, ChB, HB, π–π	[43,55,56,75,76,78]
CMB (**mod. 17**)	**52**	GAFF/AMBER	100 ns	Explicit solvents	HaB, π–π	[56]

^1^ Amylose *tris*(3,5-dimethylphenylcarbamate), ADMPC; Amylose *tris*((*S*)-α-methylbenzylcarbamate), ASMBC; Cellulose triacetate, CTA; Cellulose *tris*(3-chloro-4-methylphanylcarbamate), CCMPC; Cellulose *tris*(4-methylbenzoate), CMB; Cellulose *tris*(phenylcarbamate), CTPC; Cellulose *tris*(3,5-dimethylphenylcarbamate), CDMPC; Acetonitrile, ACN; Chalcogen bond, ChB; Electrostatic, El; Ethanol, EtOH; Halogen bond, HaB; Hydrogen bond, HB; *n*-Heptane, Hept; *n*-Hexane, Hex; Hydrophobic, Hph; Propan-2-ol, 2-PrOH; Methanol, MeOH; Noncovalent interactions, NCI; Repulsive, Rep; van der Waals, vdW. ^2^ Structure optimized based on the procedure reported by Okamoto et al. [28]. ^3^ Details on the construction of the virtual polymer are not reported in ref. [74].

## Data Availability

No new data was created or analyzed in this study. Data sharing is not applicable to this article.
